# The short-term impact of the COVID-19 pandemic on the management of private universities in Poland

**DOI:** 10.1007/s40979-023-00123-6

**Published:** 2023-02-06

**Authors:** Marcin Geryk

**Affiliations:** Jagiellonian University, Krakow, Poland

**Keywords:** COVID-19, Higher education system, Private universities, Management

## Abstract

In early March 2020, the COVID-19 pandemic changed the situation of thousands of higher education institutions around the world. The spreading virus not only imposed remote education, but it also brought tremendous changes in university management. The introduction of new educational and administrative solutions was a huge logistical and financial challenge for public and private universities. It was not easy for students either. It also posed a problem for the faculty. Remote learning generated many problems even in highly developed countries. Therefore, the number of students was expected to slowly decline. It was a surprise that private universities saw an increase in interest in their offer right after the outbreak of the pandemic. The article presents the situation of private universities in Poland, the complex problems related to the management of the academic environment in the absence of proper preparation for on-line education as well as for remote management of higher education organizations.

*Prolonged and repeated closures of education institutions are taking a rising psycho-social toll on students, increasing learning losses and the risk of dropping out, disproportionately impacting the most vulnerable. Full school closures must therefore be a last resort and reopening them safely a priority* (UNESCO 2021).

*Audrey Azoulay, Director-General of UNESCO*

## Introduction

Despite the first worrying cases of a rapidly spreading virus in central China, there were no signs that its effects would have a global impact (Worobey 2021). The first more serious data appeared on December 31, 2019, along with official information provided by the Chinese authorities to the World Health Organization (World Health Organization 2020). The World Health Organization to announce a global pandemic at the beginning of March 2020, and the new virus was called COVID-19. The scale of the epidemic threat has not had its counterpart for many last decades, hence it has affected all manifestations of human life, leading to the lockdown of virtually all manifestations of human social and economic activity (World Bank 2020).

Higher education was one of those spaces that were exposed to unprecedented challenges. In the first decade of April 2020, universities and colleges in as many as 175 countries around the world were closed. This decision meant that 220 million students would shift the learning process from traditional to remote learning (World Bank 2020).

National governments responded differently to the pandemic situation – individually assessing the time, the depth and the methods chosen. In many countries, the immediate response of the government to the need to close the campuses of post-secondary institutions were to transition as much education as possible to remote learning. Everywhere issues of equity, infrastructure, broadband capacity, and pedagogic capacity immediately emerged as challenges.

Modern technologies, present at universities for many years, allowed for remote learning. However, this form of education was not popular, both among lecturers and students. An additional problem was the rapid transition to remote learning in connection with the declaration of the COVID-19 pandemic (Hodges et al. 2020).

Disruptions and changes also touched other higher education institution activities. The COVID-19 pandemic had a huge impact on global science research (Lee and Haupt 2021). In total, 3401 articles were published on COVID-19 from January 2020 to May 2020. The top five producers were China (1079), the United States of America (624), Italy (310), the United Kingdom (209), and India (141).

The aim of the article is to present the situation of private universities in Poland during the Covid-19 pandemic. Problems related to the new principles of conducting classes and ways of dealing with them will be presented. An attempt will also be made to answer the question: has the pandemic had a positive or negative short-term impact on private universities in Poland.

## International background

Research results from the University of Birmingham and the University College London revealed that forced imposition of online learning was accepted by students. It showed that the average student assessment of the online module was 6% higher than that of the classroom-based delivery of 2019. It corresponds with an earlier study by Deloitte Access Economics which estimated that there were (in 2016) around a billion people below the age of 64 keen to learn (online) (Deloitte 2015).

A study on disruptions in United Kingdom universities was based on the perspectives of *n* = 1148 academics drawn from across a whole range of career hierarchies, the major disciplines, and different kinds of institutions. It focused on the major consequences of COVID-19 (Watermeyer et al. 2021).

After all, research shows that students, as young people, would be much more likely to accept remote learning. The same research indicates a far-reaching reserve of academic staff when it comes to using the latest technologies in the process of knowledge transfer. The resistance to such serious changes was additionally increased due to the reduced sense of security caused by the flood of information about the pandemic, which had a significant impact on shaping the attitudes of participants in the education process (Selweyn 2017; Marshall 2018; Williamson 2021).

In accordance with the recommendations of medical services, as early as March 2020, universities in the United States switched to remote forms of education, thus promoting increasing social distancing to avoid the risk of infection. Importantly, these regulations were implemented in all 50 states, which meant that 1300 colleges have been teaching online classes since then. This meant a specific test for the technical abilities of university services in the organization of technical support for the education process, as well as the ability to use these techniques by lecturers (Frazer et al. 2017).

However, the effects of these actions were different. As the research data show, 44% of universities have successfully implemented only remote education, in over 1/5 universities a hybrid model was chosen, combining the traditional form with the form of remote education, nearly 1/3 - mainly traditional education. At the same time, despite the enthusiasm declared in other studies, the majority of students claimed that remote learning would negatively affect the learning outcomes. Perhaps it was the result of the almost complete abandonment of personal meetings between lecturers and students (Lederman 2020).

The enrollment to United States universities during the pandemic was only slightly affected (the numbers were down by 3% for domestic student enrollment and 11% for international). However, more than 65% of colleges and universities started the fall 2020 semester with blended or fully online delivery (Coronavirus Hits Campus 2020). In one US survey, 72% of students wanted to return to classes in their traditional form, but a majority preferred to do so with some online components (Aleksander et al. 2020).

Another study from a public university from New Jersey, United States, brought the conclusion that social and emotional support is needed, for both students and the faculty. The learning process is a social as well as cognitive engagement and should be developed with a sense of community and resilience (Shin and Hickey 2021). The role of university managers was deeply changed. They had been prepared to work in normal conditions, not in a global pandemic, so crisis leadership received high attention in the higher education leadership literature (Ruben 2020).

A survey of 172 department chairs in the United States emphasizes the academic chairperson role, which underlines the need for increased training and development to reinvent institutions of higher education (Gigliotti 2021). Another issue was raised in the United Kingdom, where from September 2020 most lectures in United Kingdom universities were delivered online.

Similar aforementioned situation took place in private universities. In Germany, private universities also struggled during the Covid-19 pandemic. According to the results of a survey, a third of the universities observed a decline in international students’ enrollment and a drop of funding from donors. Their financial situation reached a perilous level, according to 41% of respondents. Nevertheless, the (Stifterverband 2022) pointed out that, according to student surveys, the quality of teaching at private universities was still higher during the pandemic than at state universities (Boytchev 2021).

In New Zealand, universities were exposed to the risk of losing up to New Zealand dollars (NZD) 300 million due to the government travel ban caused by COVID-19. This restriction kept all 6500 students, who were enrolled this year, at home (Campbell 2020).

Taking a stakeholder perspective, also by converting all the activities, i.e. lectures, exams, laboratories, support services, into an online form quickly and effectively was the main objective since the very beginning. Overall, the main message could be summarized as ‘together, we can make it’. At the national level, in Italy, the funds provided to higher education institutions gave them the possibility to make large technological investments by increased research funding, through increased scholarships, researcher positions and a national research plan to sustain the economic recovery (Agasisti and Soncin 2021).

In Australia, the federal government insisted that universities expand the short courses offer as well as new trainings in certain jobs as national priority. The disruptions in the system of higher education caused by COVID-19 were seen there as a huge opportunity for a strategic transformation in universities towards being more differentiated in character and missions. Remote learning and digital tools helped to create the hybrid mode of learning and to develop learning skills of the staff (Bebbington 2021).

In Hongkong (S.A.R.) many universities shifted from the model that is physically open to society and students and displays strong intellectual dynamism to one that is controlled, closed, empty, and allows for little intellectual engagement. Concerns were also raised over career-planning, for both students and younger academics. It all led to the situation where only 26% of students were satisfied with their remote learning experience; more than 60% thought that the learning effectiveness of online courses was poorer than that of face-to-face courses, questions were raised about the future of knowledge creation and higher education in general (Xiong et al. 2020).

In the Republic of South Africa, the first governmental reaction to COVID-19 was the reduction of the budget for higher education by 20% (ZAR19 billion). This reduction had a crucial impact on 26 public universities that accounted for 85% of university enrolments in 2017 (Department of Higher Education and Training (DHET) 2019). One study reported that 46% of students faced challenges in completing assignments or participating in remote learning due to lack of access to computer equipment or data (Jordaan 2020). It only showed that the problem of inequality and lack of funds made the pandemic situation even worse than in other countries. As the former vice-chancellor of the University of Witwatersrand, Adam Habib, said: “South African universities have similar problems to other institutions across the world. The big distinction with South Africa is that we are undertaking these activities in the midst of deep inequalities” (Habib et al. 2020).

Digitalization of universities is widespread around the world. Usage of electronic platforms, for online teaching, research, making campuses ‘smart’ are only a few examples of those actions. Providers like Online Programme Management (OPM) and Massive Open Online Course (MOOC) help to deliver programs online. As of September 2020, there were 20 identified EdTech unicorns in the world, i.e., companies valued over $1bn (The list of EdTech unicorn companies is provided by Holon IQ 2022). Out of those, 17 are from China and the United States of America. These two countries are dominating the digital economy more broadly and are indicative of the new power struggles in the global economic order (United Nations Conference on Trade and Development (UNCTD) 2019). It only shows that digitalization of higher education is bound with a broader expansion of the digital economy. So, data privacy becomes a huge concern as well as the processes of assetisation (Birch and Muniesa 2020) in close connection to the theory of rentiership (Birch et al. 2020).

In New Zealand, the closing of the country’s borders to foreign students led to the risk of a decline in revenues by 300 million NZD and prevented the arrival of more than 6500 students from China (Campbell 2020).

In Poland, as in many other countries worldwide, along with the growing anxiety in the face of the pandemic, universities began to close at the beginning of March 2020 (University World News 2020). Until the end of the 2019/2020 academic year, higher education institutions carried out remote education with the use of remote learning tools. A similar solution was also applied in the 2020/2021 academic year. The regulations introduced in the spring of 2020 radically changed the functioning of all academic institutions, including private universities. The latter, due to their almost total dependence on revenues from tuition fees, understandably began to worry about an uncertain future.

Research conducted in September 2020 by the Center for Education and Development “Efekty” on a group of 135 universities (82% of which are representatives of state universities, and 18% - private ones) from the largest academic centers in Poland (Warsaw, Kraków, “Tri-City” (three cities in northern Poland - Gdansk, Gdynia and Sopot), Upper Silesian Agglomeration, Poznan and Wroclaw) shows that after the experience of the summer semester of the 2019/2020 academic year, which was conducted remotely, 85% planned to organize the new academic year (2020/2021) in a remote and/or hybrid form. At the same time, two thirds of them recognized that they are prepared for this type of education.

Notably, representatives of the private universities participating in the study did not see a major negative impact of the pandemic on admission levels. They predicted, however, that promotion expenses would have to be increased, and the marketing activity itself would take place almost exclusively on the Internet (Centrum Edukacji i Rozwoju ‘Efekty’ 2021).

It thus appears that the difficulties which imposed the changes in the existing management practices proved to be more of a challenge than an obstacle to further development of universities in Poland. Certainly, the most difficult element of this process was the sudden shift of higher education institutions to a different mode of contact with students in terms of internal and external functioning of the organization itself.

In fact, the main concern among private universities was the presumption of how deep the impact might be on candidates’ decisions about enrolling to higher education institution where tuition fee is required. It was assumed that along with the decline in labour market activity and the loss of livelihoods by a certain number of people, the number of students would drop significantly, deteriorating the already unfavourable situation of the private university sector. Fortunately, private universities are sending quite a different message. The largest of them record increased interest and recruit over 10 % more candidates than in previous academic years. But it is difficult to predict whether this trend is permanent or whether the threat of the growing scale of problems among colleges has only been postponed and will explode with greater force in the coming years (Polska Agencja Prasowa 2020).

## The situation on the private universities market in Poland

The Polish education system has undergone profound changes since the political transformation in 1989 (the collapse of the communist system in Poland) concerning the curricula, structures, organization, and management. As noted by Marek Kwiek, starting from 1989, the system experienced “a phenomenal increase in the number of state and private institutions and an increase (and then decrease) in the number of students” (Kwiek 2015). In 1991, the first private universities were established in Poland, which now boast almost a thirty-year history of operation. After the initial boom in the sector, the greatest intensity of which occurred in the first two decades, especially in the dynamic years 1990–1999 (Geryk 2007), a decline in the number of private universities began. While between 2008 and 2010 there were about 326 such institutions in Poland, in 2013 there were only 301, and in 2016 just 252. The latest data from the Central Statistical Office (for 2019) show that there are 221 private universities. This means that in the last decade, i.e. between 2009 and 2019, the number of private universities in Poland decreased by 32.6%, which means that every third university in this sector was closed or consolidated with another centre (GUS 2020).

Such a strong decline in the number of universities was a direct consequence of both demographic changes and the dwindling number of candidates. This problem became acutely felt in the whole of higher education and especially in the private sector, which offers paid tuition. Over the period of 2009–2019, the decline in the number of students was even more dramatic than the decline in the number of universities, amounting to 46%. In absolute numbers, this means that in 2009 over 630,000 students attended private universities, and in 2019 – only 340,000. By far the most adverse effects in this respect were observed between 2011 and 2013, when the number of students decreased by 11–13% each year. While the aforementioned downward trend in the number of private universities has continued since 2010 until now, the downward trend in the number of people attending these institutions came to a halt in 2017, and the next 2 years brought the first increase in the number of students in over a decade. In 2019 private universities educated about 6.1% more students than in 2017, and increased recruitment could be observed in 13 out of 16 voivodships (GUS 2020).

A constantly growing number of foreign students is a very characteristic trend among private universities. Between 2009 and 2019, the number of foreign learners increased almost ninefold, i.e. from 4.4 thousand to 39.5 thousand. The share of foreign students in the total number of students in private universities increased from 1% to 11.5%. Changes in this respect concern the entire country, and not only its border regions (Tomczyńska et al. 2021).

The changes that have been taking place have enforced and still enforce a positive selection in the sector, leading to closures of universities that are not profitable or offer low-quality services. Despite the strong transformations that the private university sector has experienced in the past decade, it still represents a very important element of the higher education system in Poland.

## Research methodology

In the summer semester of the 2019/2020 academic year, universities, like many other institutions, were forced to reorganize their activities quickly and had to adapt to the requirements of the suddenly widespread epidemiological situation.

It was necessary not only to transfer the didactic activity to the virtual world, but also to create new rules for research and scientific work, for administrative activities and for the organisation of tests and exams. Those tasks turned out to be significantly more demanding as there was no time to prepare for them in advance. Due to the occurrence of such an unusual situation, it was decided to create a questionnaire on issues related to the epidemiological situation, and more specifically - in order to assess the impact of the pandemic on the functioning of universities and the degree to which they successfully deal with this challenge. Due the uniqueness and individuality of these studies, it was decided to present their results in this study. In addition, questions were also asked about the quality of actions by state authorities and about the expectations of universities towards such bodies as the ministry or the government.

The research conducted by the author in the period between May and September 2020 was done using the CAWI (Computer-Assisted Web Interview) technique. CAWI, or Computer Assisted Web Interview is an interview conducted via an Internet channel. The chosen respondent receives and completes the survey provided to him or her through the same online channel. It seemed like an obvious technique during pandemic period of COVID-19 and when respondents were available online only.

Invitations to participate in the survey, along with the relevant link, were sent by e-mail to all private universities in the POL-on database (Integrated Information Network on Science and Higher Education), 219 institutions altogether. The request to fill in the questionnaire was addressed to the authorities of individual universities, in particular to rectors, vice-rectors and chancellors, but it was also possible for the authorities to appoint another university employee with extensive knowledge about its functioning. Due to the restrictions related to the COVID-19 pandemic and the transition to the remote work system, it was necessary to refrain from supplementing the CAWI technique with direct contact with respondents. After a several months’ effort, 41 questionnaire interviews with private university representatives were completed. Which was not such an insufficient response given the conditions of the first lockdown and regarding the organizational issues concerning remote learning and additional online duties.

The main objective of the study was to try to determine the actual impact of the COVID-19 pandemic on university management. The aim was therefore to assess the extent to which the current practice of management was deformed by a sudden change of the functioning of private universities in Poland.

## Results

**Fig. 1:**
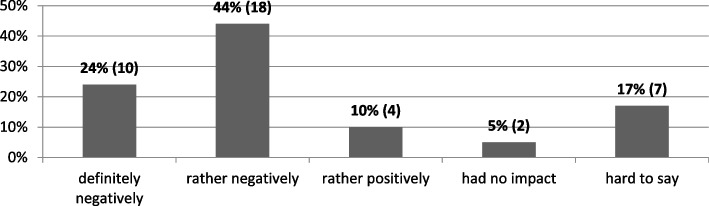
How did the coronavirus pandemic situation affect the overall situation of the private university sector in Poland? (*N* = 41). Source: author’s own study

Several months after the outbreak of the COVID-19 pandemic, representatives of most of the universities who took part in the survey agreed that the situation had had a negative impact on the private university sector. This opinion was expressed by 68% (28) of respondents, including 24% (10) who agreed strongly. These changes were positively assessed by 10% (4) of the respondents, and 5% (2) said that the epidemic did not affect the overall situation of the sector. The remaining others did not have an explicit opinion on the subject (Fig. 1).

**Fig. 2:**
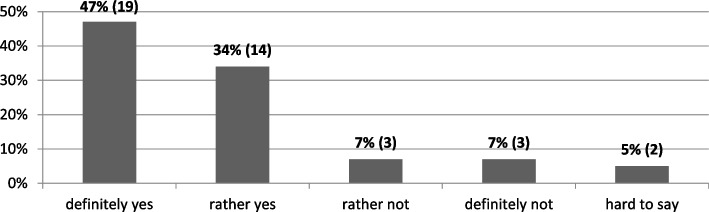
Is managing a private university in the coronavirus pandemic conditions more difficult than it was in the last few years? (*N* = 41). Source: author’s own study

More than 80% (> 33) of private university representatives surveyed agreed that due to the pandemic managing this kind of institutions became more difficult than in recent years. Interestingly, as many as 47% (19) expressed this view in a decisive manner. Only 14% (6) of the respondents did not register any negative changes in this respect (Fig. 2).

**Fig. 3:**
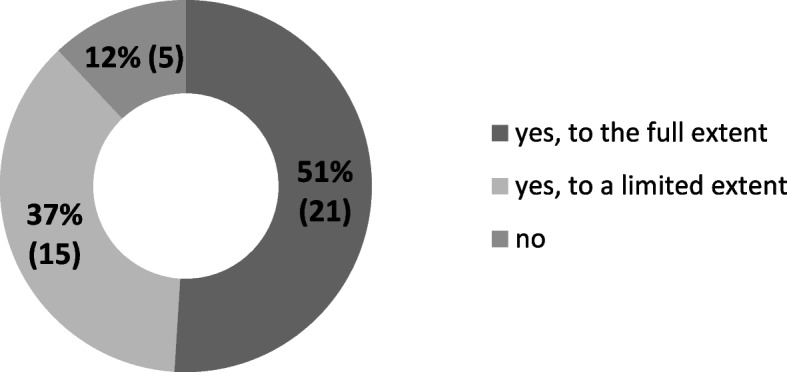
Was your university prepared for distance learning? (*N* = 41). Source: author’s own study

Based on the self-assessment submitted by representatives of private universities, it can be concluded that half of them were prepared to implement remote learning in its full extent. At the same time, 37% (15) of these types of institutions pursued this goal in a narrow scope, i.e. only in relation to specific fields of study, subjects or groups of students. Representatives of 12% (5) of the universities felt that their centres were not at all prepared for the transition to remote learning (Fig. 3).

**Fig. 4:**
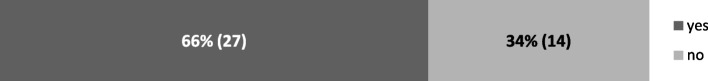
In connection with the pandemic outbreak, was a special team appointed at your university to adapt the university to the new operating conditions and to minimize the negative effects of the situation? (*N* = 41). Source: author’s own study

Due to the occurrence of the pandemic, two-thirds of the universities appointed a special team designed to bring their universities to work in the new conditions and to adapt them to the changing sanitary requirements and restrictions (Fig. 4).

**Fig. 5:**
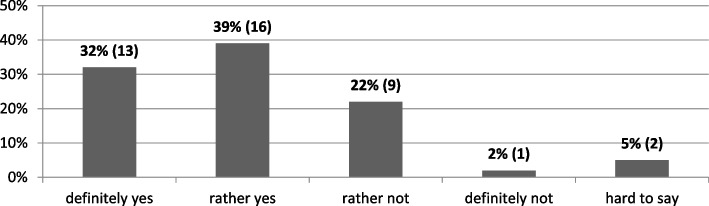
Do you think the coronavirus pandemic may have a negative impact on student recruitment for the next academic year due to the limited possibilities of university promotion? (*N* = 41). Source: author’s own study

More than 70% (> 29) of university representatives feared that the situation related to the coronavirus pandemic may have a negative impact on the student recruitment for the 2020/2021 academic year, for example due to the limited possibilities of university promotion (cancelled fairs, open days, promotional meetings in secondary schools). 24% (10) of the respondents were of the opposite opinion (Fig. 5).

University officials were asked to assess how their facility had dealt with the various challenges that emerged with the coronavirus pandemic situation. The results are very positive: in the context of many activities there were no negative assessments, and where such occurred, they did not exceed the level of 10% (4). Representatives of all private universities participating in the study assessed their activities positively (there were no “rather badly” or “very badly” responses) in such areas as:

- development of guidelines (procedures) on how to conduct remote learning,
- adapting the working conditions of administrative and technical staff to the applicable sanitary requirements (keeping a distance, using masks and gloves, disinfecting rooms, etc.),
- issuing certificates, diplomas and other documents related to the course of study for students in electronic form,
- accepting applications, diploma thesis and other documents related to the course of study from students in electronic form,
- the recruitment process,
- enabling administrative staff to work remotely.

**Fig. 6:**
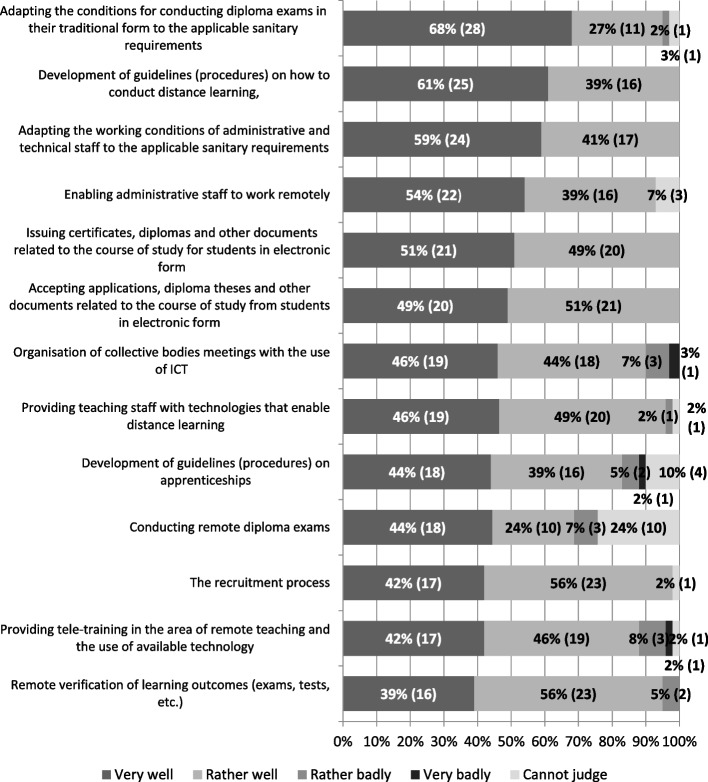
How did your university deal with the following tasks in the COVID-19 pandemic situation? (*N* = 41). Source: author’s own study

Organisation of remote meetings of collective bodies, using information technology, and the provision of access to tele-training on the use of remote learning tools were a source of most problems. Representatives of 10% (4) of the universities declared that their institution had coped poorly with these tasks (Fig. 6).

**Fig. 7:**
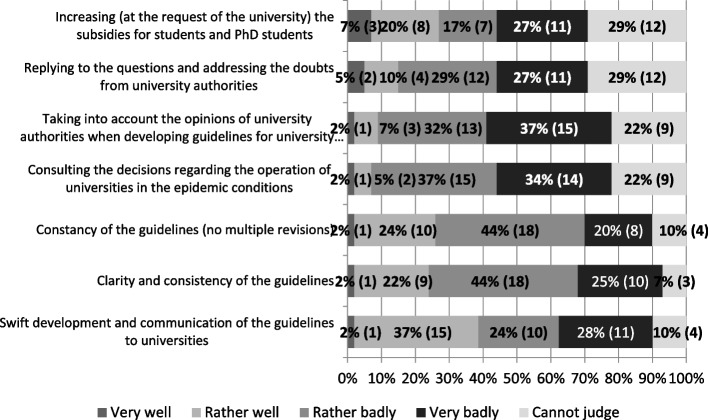
Assessment of actions by state authorities (the government, Ministry of Science and Higher Education) aimed at private universities in connection with the outbreak of the coronavirus pandemic (*N* = 41). Source: author’s own study

Representatives of the private higher education sector assessed the actions of state authorities (the government and the Ministry of Science and Higher Education) aimed at universities in connection with the pandemic rather negatively. About 70% (28) of the universities negatively assessed the activities of the authorities in terms of how they consulted their decisions or whether actually took into account the opinions of private universities when developing the guidelines. 7% to 9% (3 to 4) of universities assessed this area positively. The clarity and coherence of the guidelines (69% – 28 of negative evaluations) and their stability (64% – 26 of negative evaluations) were often assessed negatively. However, both areas were positively assessed by every fourth university (24%–26% – 10). Of all the aspects of the authorities’ response to the crisis situation the speed of developing and providing universities with guidelines for their functioning in the COVID-19 pandemic conditions were assessed the most favourably (Fig. 7).

**Fig. 8:**
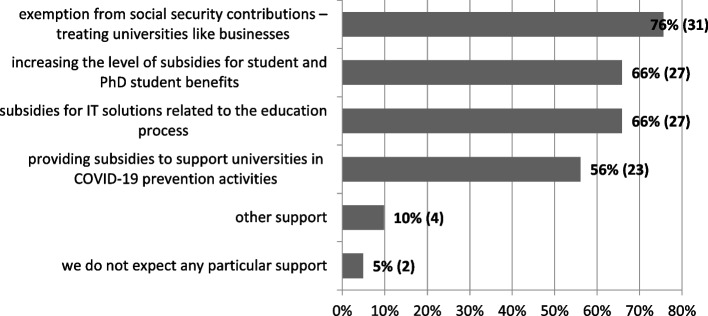
What support does your university expect from state authorities in relation to the pandemic outbreak (*N* = 41, multiple choice). Source: author’s own study

When asked whether the university expects support from state authorities in connection with the COVID-19 pandemic, only 5% (2) of the respondents gave a negative answer. Representatives of the other universities expected, primarily, exemption from the obligation to pay social security contributions, similarly to other business entities (76% – 31). In the second place was the need to co-finance IT solutions necessary for education and an increase in subsidies for student and PhD student benefits (66% – 27 each). More than half of the universities considered it justified to grant subsidies for activities related to COVID-19 prevention, including, for example, the purchase of disinfectants (Fig. 8).

## Discussion and conclusion

The difficulties in the functioning of universities are partly a reflection of the huge shock caused by the pandemic. The sudden transition of the entire higher education system to the remote teaching mode forced universities not only to undertake many organizational projects, but above all led to increased expenditure, for example on IT infrastructure. Over two thirds of the respondents participating in the study confirmed the negative impact of the pandemic on universities, and as many as four fifths of them emphasised the difficulties in managing a university in such a situation. Universities were rather efficient in establishing appropriate collegial bodies for the purposes of introducing a sanitary regime. The fact that a vast majority of them were prepared for remote education and work should also be assessed positively – only 12% (5) of the surveyed universities did not manage to rise to this challenge.

Relations with state authorities were assessed negatively. About 70% (29) of the respondents indicated lack of consultation on university related regulations. Even though the survey concerned private universities, only 5% (2) of their representatives did not expect any financial support from the state. It appears therefore that private universities perceive their sector as an important element of the higher education system. This corresponds inversely to the extensive state aid granted to private entities in other sectors of the economy.

The results of the research clearly show the high adaptability of the Polish sector of private universities, which, it must be emphasized, functions practically without any financial support from the state. The respondents critically indicated that even in the pandemic situation, this help was practically illusory.

In general, one might say that a strong negative impact of the pandemic and lockdown regulations were observed among private universities. The vast majority described their level of preparation for such a challenge as high. The most problematic issue was the process of verification of learning outcomes. By and large, one may state that the COVID-19 pandemic caused a certain shock. However, after a difficult initial period, especially at the turn of the first and second quarter of 2020, universities began to deal with the remote implementation of the teaching process and statutory tasks much more efficiently.

## Limitations

The research was carried out in the first year of the COVID-19 pandemic and in the first few months of the restrictions (lockdown), i.e. in the period May–September 2020. Universities had not yet had much experience in remote learning, both in terms of the organization of remote work, assessment quality of education and checking the effects of this process. Certainly, the pace and scope of the introduced changes had an impact on the results of the study. On the basis of the obtained results, it is debatable to assess to what extent they are a manifestation of the changes themselves, and to what extent they are an important factor of influence, which was the widespread fear for health, intensified by intensive media coverage.

At the same time, one should be cautious about the degree of definiteness of the respondents’ statements, as they refer to the first few months of functioning in a changed reality. Another limitation is the number of obtained questionnaires - only 45 out of 219 non-public universities in Poland to which the questionnaires were addressed.

It seems that further research should be continued, both to broaden the degree of representativeness of the results and to confirm or contradict the statements contained in the article.

## Data Availability

The data that support the findings of this study are available from the first and sole author (M.G.) upon reasonable request.

## Abbreviations

CAWI: Computer-Assisted Web Interview
OPM: Programme Management
MOOC: Massive Open Online Course
NZD: New Zealand dollars
